# Exploring susceptibility to atrial and ventricular arrhythmias resulting from remodeling of the passive electrical properties in the heart: a simulation approach

**DOI:** 10.3389/fphys.2014.00435

**Published:** 2014-11-12

**Authors:** Natalia A. Trayanova, Patrick M. Boyle, Hermenegild J. Arevalo, Sohail Zahid

**Affiliations:** Department of Biomedical Engineering, Institute for Computational Medicine, Johns Hopkins UniversityBaltimore, MD, USA

**Keywords:** arrhythmia, computer modeling, structural remodeling, fibrosis, infarct

## Abstract

Under diseased conditions, remodeling of the cardiac tissue properties (“passive properties”) takes place; these are aspects of electrophysiological behavior that are not associated with active ion transport across cell membranes. Remodeling of the passive electrophysiological properties most often results from structural remodeling, such as gap junction down-regulation and lateralization, fibrotic growth infiltrating the myocardium, or the development of an infarct scar. Such structural remodeling renders atrial or ventricular tissue as a major substrate for arrhythmias. The current review focuses on these aspects of cardiac arrhythmogenesis. Due to the inherent complexity of cardiac arrhythmias, computer simulations have provided means to elucidate interactions pertinent to this spatial scale. Here we review the current state-of-the-art in modeling atrial and ventricular arrhythmogenesis as arising from the disease-induced changes in the passive tissue properties, as well as the contributions these modeling studies have made to our understanding of the mechanisms of arrhythmias in the heart. Because of the rapid advance of structural imaging methodologies in cardiac electrophysiology, we chose to present studies that have used such imaging methodologies to construct geometrically realistic models of cardiac tissue, or the organ itself, where the regional remodeling properties of the myocardium can be represented in a realistic way. We emphasize how the acquired knowledge can be used to pave the way for clinical applications of cardiac organ modeling under the conditions of structural remodeling.

## Introduction

Computer modeling has emerged as a powerful platform for the investigation of lethal heart rhythm disorders. Biophysically detailed simulations can explain experimental observations and help reveal how organ-scale arrhythmogenic phenomena (ectopic heartbeats, conduction failure, electrical turbulence, etc.) emerge from pathological effects at the tissue, cell, and protein levels. The development of this extensive “virtual heart” methodology (Noble, 2002; Vigmond et al., 2009; Gurev et al., 2011; Trayanova, 2011; Winslow et al., 2012) builds upon a strong foundation of research that seeks to use experiments and simulation to quantitatively characterize the action potential response of cardiac cells to electrical stimuli. Recent advancements in single-cell action potential modeling have produced building blocks for constructing models of the atria (Courtemanche et al., 1998; Nygren et al., 1998; Grandi et al., 2011), the ventricles (Ten Tusscher and Panfilov, 2006; Fink et al., 2008; Grandi et al., 2010; O'Hara et al., 2011), and the cardiac conduction system (Stewart et al., 2009; Aslanidi et al., 2010; Sampson et al., 2010; Li and Rudy, 2011; Vaidyanathan et al., 2013) with unprecedented levels of biophysical detail and accuracy. Such developments have helped to fuel the exciting progress made in simulating cardiac electrical behavior at the organ level (Moreno et al., 2011; Tandri et al., 2011; Trayanova et al., 2012; Boyle et al., 2013, 2014; Hu et al., 2014; Trayanova and Boyle, 2014; Clayton and Bishop, in press). Emergent, integrative behaviors in the heart result not only from complex interactions within a specific level but also from feed-forward and feedback interactions that connect a broad range of hierarchical levels of biological organization.

Several recent reviews have been written on our current understanding the mechanisms of atrial and ventricular mechanisms from an integrative interactions perspective (Janse, 2004; Rubart and Zipes, 2005; Jacquemet et al., 2008; Rudy et al., 2008; Plank et al., 2008b; Fishman et al., 2010; Dossel et al., 2012; John et al., 2012; Trayanova, 2012, 2014; Chen et al., 2014; Heijman et al., 2014), often derived from computer simulations, however, most of these focus on how remodeling in the active electrophysiological properties of the diseased heart contributes to the increased propensity to arrhythmias. One aspect of cardiac tissue behavior that has received less attention is the contribution, to the initiation and maintenance of arrhythmias, of the changes in the “passive” tissue properties that take place in the diseased heart. Under “passive” we refer to the aspects of electrophysiological behavior that are not associated with active ion transport across cell membranes; these include current flow in the intra- and extracellular domains of cardiac tissue and the aspects of tissue composition and structure that determine the direction and magnitude of these currents. Under diseased conditions, remodeling of the passive tissue properties takes place, most often resulting from structural remodeling such as gap junction down-regulation and lateralization, fibrotic growth infiltrating the myocardium, or the development of an infarct scar. Such structural remodeling renders atrial, or ventricular tissue as a major substrate for arrhythmias. The current review focuses on these aspects of cardiac arrhythmogenesis.

However, because of this inherent complexity of cardiac arrhythmias, it is often difficult to dissect the contributions of individual players, and to elucidate interactions at a particular spatial scale. Computer simulations of cardiac electrophysiology have provided this ability. In this article, we review the current state-of-the-art in modeling atrial and ventricular arrhythmogenesis as arising from the disease-induced changes in the passive tissue properties, as well as the contributions these modeling studies have made to our understanding of the mechanisms of arrhythmias in the heart. Because of the rapid advance of structural imaging methodologies in cardiac electrophysiology, we chose to present studies that have used such imaging methodologies to construct geometrically realistic structural models of cardiac tissue, or the organ itself, where the regional remodeling properties of the myocardium can be represented also in a realistic way. We emphasize how the acquired knowledge can be used to pave the way for clinical applications of cardiac organ modeling under the conditions of structural changes. This review does not intend to be exhaustive on the subject, but to provide examples of how computer modeling could be instrumental in understanding arrhythmogenesis as it arises from disease remodeling at the various levels of biological and structural organization of the heart.

## Overview of methodology for simulating arrhythmogenesis and representing the remodeling in the passive properties of the myocardium

**Figure 1:**
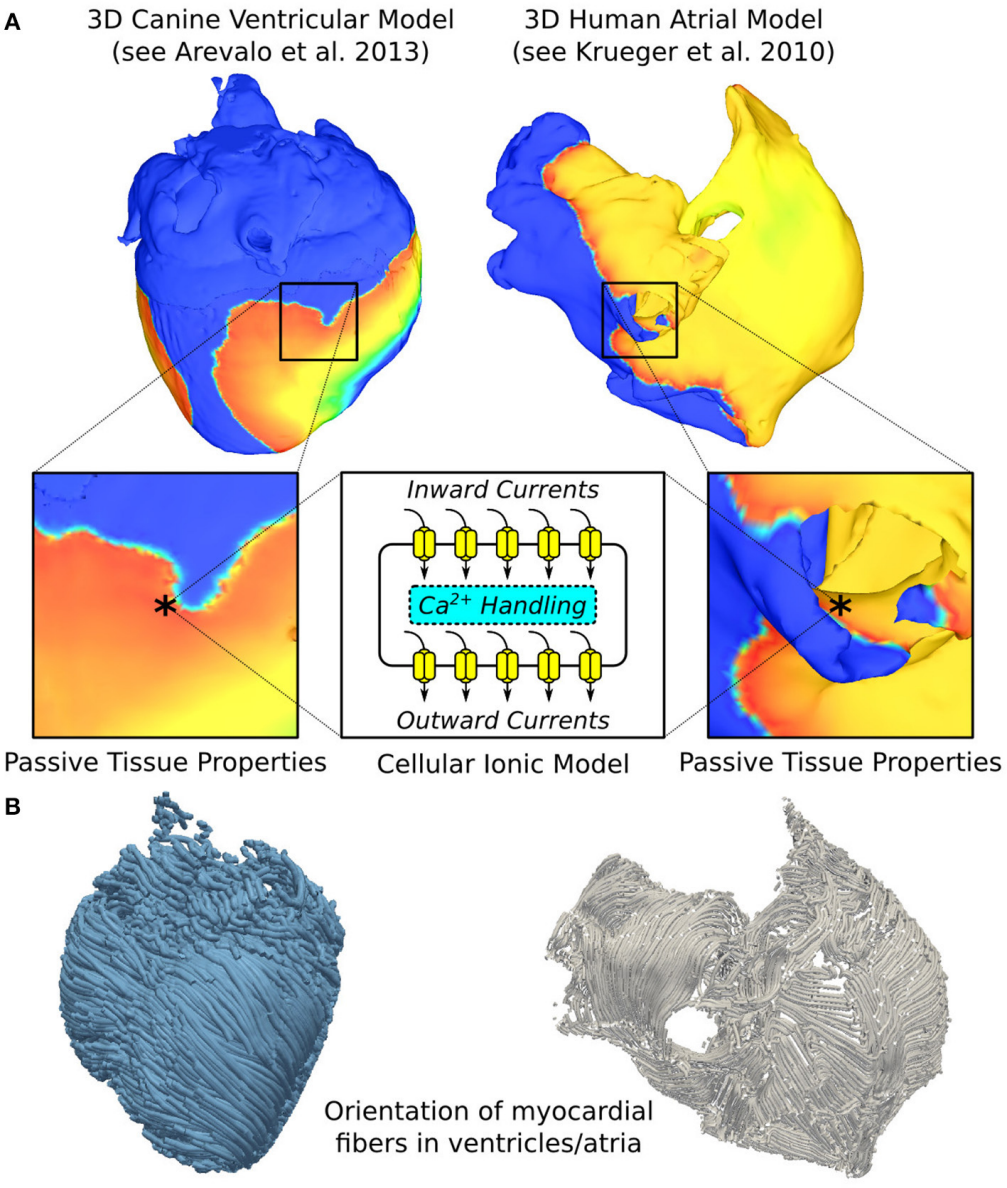
**Overall approach to image-based modeling of cardiac electrophysiology**. **(A)** The multiscale aspect of cardiac electrophysiological models. Passive electrical coupling of cardiac cells mediates the tissue-scale propagation of bioelectric impulses that originate at the membrane level (action potentials). ^*^indicates representative points in ventricular and atrial tissue models. 3D geometrical models are reconstructed from images of a canine heart (left), as in Arevalo et al. (2013), and human patient atria (right), as in Krueger et al. (2011). **(B)** Schematics showing the orientation of myocardial fibers in the ventricular and atrial models. Left panel modified with permission from Arevalo et al. (2013).

Computer modeling of arrhythmogenesis has made enormous progress over the last decade, enabling the simulation of electrical function in cardiac tissue as well as in the entire organ. A schematic of the current state-of-the-art general approach to 3D multi-scale (from the molecule to the organ) arrhythmia modeling (atrial or ventricular) is shown in Figure 1A. Ionic exchanges across cell membranes, via ionic channels, pumps, and exchangers, represented by an action potential ionic model comprising of numerous ordinary differential and algebraic equations, drive current flow in the tissue. In the multi-scale atrial or ventricular model, propagation of the wave of action potential is simulated by solving (Vigmond et al., 2002, 2003; Plank et al., 2008b) a reaction-diffusion partial differential equation describing current flow through tissue composed of myocytes that are electrically connected via low-resistance gap junctions. Cardiac tissue has orthotropic passive electrical conductivities that arise from the cellular organization of the myocardium (cardiac muscle) into fibers and laminar sheets. Global conductivity values in the atrial or ventricular model are obtained by combining fiber and sheet organization with myocyte-specific local conductivity values.

Local fiber directions are typically mapped based on histological sectioning information (Nielsen et al., 1991; Vetter and McCulloch, 1998), or on diffusion tensor (DT) magnetic resonance imaging (MRI) (Helm et al., 2005), sometimes using an atlas heart (Vadakkumpadan et al., 2012). Figure 1B, left panel presents fiber orientation, as reconstructed from DTMR images, in the canine ventricles shown in Figure 1A. In cases where neither histological nor DTMR imaging information is available, rule-based approaches have been used to assign fiber orientation consistent with measurements, either manually or using a semi-automatic rule-based approach (Krueger et al., 2011; Bayer et al., 2012; Dossel et al., 2012). This particularly applies to atrial fiber orientation (Figure 1B, right panel) since DT imaging of the thin atrial walls does not provide reliable information about atrial fiber architecture.

The passive electrical properties of the myocardium (Figure 1A) can be regionally remodeled in disease, with the regional change originating from several major sources: (1) changes in gap junction resistance between cells resulting in changes in conductivity values; (2) deposition of collagen forming either local resistive barriers between fibers or replacing myocytes (interstitial or replacement fibrosis; in the atria, it is a hallmark of the aging tissue); (3) formation of an infarction scar in the ventricles as a result of ischemic cardiac disease; and (4) abnormal proliferation, under diseased conditions, of non-myocyte cells, such as myofibroblasts, which may or may not interact with the cardiomyocytes electrically.

**Figure 2:**
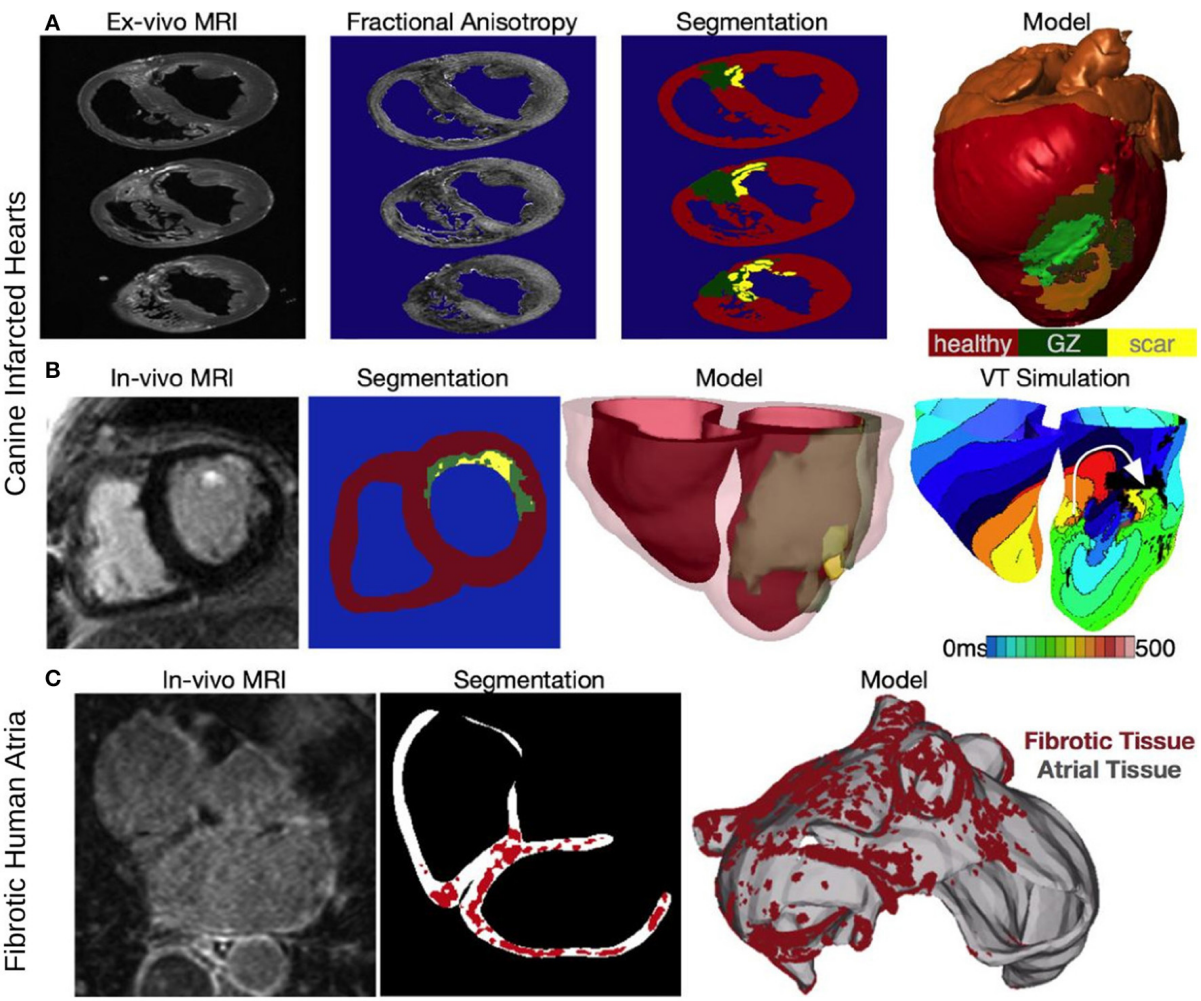
**Constructing image-based models of the ventricles from an infarcted canine heart (A,B) and the fibrotic atria of a human patient with AF**. **(A)** Reconstruction from an *ex-vivo* MRI scan of an infarcted canine heart. Fractional anisotropy (FA) maps as calculated from the DT-MRI, brighter color corresponds with higher FA value. The images are segmented into healthy myocardium, gray zone (GZ), and scar, to reconstruct an image-based model of the infracted canine heart (right-most panel). Modified with permission from Arevalo et al. (2013). **(B)** Construction of a patient-specific ventricular model of arrhythmia from a clinical MR scan. Shown are images of an infarcted patient heart before ablation (treatment) and the corresponding segmentation: healthy (red), GZ (green), or scar (yellow). An image of the three-dimensional geometric model of the patient heart rendered with the epicardium and the infarct border zone semitransparent is shown in the third panel. The right-most panel presents *in silico* activation map of arrhythmia, revealing reentry on the left ventricular endocardium. The color code in the bottom right shows electrical activation time. Modified with permission from Winslow et al. (2012). **(C)** A model of the fibrotic human atria generated from a patient LGE-MRI scan (top left) following segmentation (top right) into normal and fibrotic tissue (fibrotic lesions in red). With permission from McDowell et al. (2012).

Multi-scale models of arrhythmias are typically modular, allowing the use of any cellular ionic models, of different species and with different levels of biophysical detail. Furthermore, solutions are executed on user-specified organ geometries, which can be idealized, or anatomically-accurate, the latter either representing averaged geometries obtained from histological sectioning (Nielsen et al., 1991; Vetter and McCulloch, 1998), or individual hearts' (atria and/or ventricles) geometry, and structure (Bishop et al., 2010; Vadakkumpadan et al., 2010). Using MRI data for model geometry is essential in represent individual hearts' structural remodeling in the passive tissue properties, such as ventricular infarction or atrial fibrosis. Figure 2A presents the generation of the geometry/ structure of a whole-heart model of canine infarction from high-resolution *ex-vivo* MRI scans, where a level set method was applied to the MRI image stack to separate the myocardium from the surrounding suspension media. The infarct was segmented out into the two infarct zones, scar and the electrically-remodeled border zone, also termed gray zone (GZ) based on it appearance in the MRI scans; to do so the combined information from both the DTMRI, and the structural MRI scans was used (via the calculation of fractional anisotropy, Figure 2A). The resulting infarct segmentation revealed strands of GZ tissue penetrating the electrically inert scar tissue (segmentation panel, Figure 2A). The reconstructed whole heart canine model is shown in Figure 2A with the locations of different remodeling regions combined with the organ geometry information.

Clinical MRI scans with a contrast agent (late gadolinum enhancement, LGE, MRI) can also be used to visualize the structural remodeling in atria and ventricles (Nazarian et al., 2005; Assomull et al., 2006; Oakes et al., 2009; Roes et al., 2009; Akoum et al., 2011). Figure 2B presents ventricular model generation from clinical LGE-MRI images, as described in a recent paper (Prakosa et al., 2014). Finally, Figure 2C illustrates structural changes in a patient atrium, as reconstructed from clinical LGE-MRI scans, and described in detail in the original papers (McDowell et al., 2012, 2013) It has to be noted, however, that the segmentation of the LGE MRI fibrotic regions and even segmentation of the geometry of the thin atria from clinical MRI is fraught with uncertainty and an area of intense image-processing research.

Figure 2B, right-most panel, demonstrates how the images can be used to examine susceptibility to arrhythmias of the substrate and specifically, the role of structural remodeling (and thus remodeling in the passive electrical properties). It shows the generation of a reentrant arrhythmia in the infracted patient ventricles. Numerical approaches for simulating the electrical behavior of the heart have been described in detail in previous publications, some of which offer comprehensive reviews on the subject (Rodriguez and Trayanova, 2003; Jacquemet et al., 2008; Plank et al., 2008b; Trayanova, 2011; Dossel et al., 2012).

## Fibrotic remodeling in the atria and its contribution to atrial fibrillation

Structural remodeling, and specifically fibrosis, has been associated with the persistent/permanent version of atrial fibrillation (AF) (Burstein and Nattel, 2008; Yue et al., 2011). Fibrotic remodeling of atrial tissue involves processes that occur in parallel across multiple scales: at the membrane level, gap junction remodeling due to connexin 43 (Cx43) protein downregulation/hypophosphorylation, and lateralization (Kostin et al., 2002; Burstein et al., 2009), at the cellular level, fibroblast proliferation and phenotype switching (Rohr, 2009; Yue et al., 2011), and at the tissue level, the deposition of excess collagen (Xu et al., 2004; Burstein and Nattel, 2008), both from reactive interstitial fibrosis separating muscle bundles, and from reparative fibrosis replacing dead cardiomyocytes, both interfering with electric continuity and slowing conduction (Xu et al., 2004; Iwasaki et al., 2011). Thus, structural remodeling, combined with remodeling at the ion channel level, gives rise to complex interactions at the organ level, setting the stage for AF initiation and maintenance in the fibrotic atria.

Models of the fibrotic atria have accounted for different aspects of fibrotic remodeling, in an attempt to elucidate the mechanisms leading to altered conduction and those responsible for the drivers and organization of permanent AF. The simplest model representation of atrial structural remodeling was based on the assumption that a component of structural remodeling, gap junction remodeling (Cx43 downregulation/hypophosphorylation, and lateralization), occurs throughout the atria in a uniform fashion. Two such studies have been conducted thus far: one assumed that the coupling strength between computational cells was decreased (Cx43 downregulation/hypophosphorylation only) (Krogh-Madsen et al., 2012), while the other modeled increased anisotropy throughout the LA (representing both aspects of Cx43 remodeling) (Plank et al., 2008a). One of these simulation studies showed (Krogh-Madsen et al., 2012) that decreasing the coupling between cells slowed conduction and decreased the wavelength, further perpetuating AF. The other study (Plank et al., 2008a) demonstrated that increased anisotropy throughout the fibrotic human LV was an additional mechanism for the breakup of PV ectopic waves into multiple reentrant circuits; higher anisotropy ratios resulted in sustained reentrant activity even though the ectopic focus was no longer present. Similar conclusions were obtained from a human atrial model (Krueger et al., 2014) where the locations of the fibrotic (i.e., high-anisotropy-ratio) regions were implemented from patient MRI-LGE scans.

The next component of fibrosis, collagen deposition, has been represented in models as insulating barriers, and in several ways: (i) by removing randomly the electrical connections between two 2D layers of atrial tissue, the endocardial and the epicardial, in order to model an increased level of dissociation between these two layers (a form of reactive interstitial fibrosis), mimicking experimental observations in goats (Eckstein et al., 2011); (ii) by introducing a set of random collagenous septa disconnecting cardiac fibers in the transverse direction (Jacquemet and Henriquez, 2009) (reactive interstitial fibrosis again); and (iii) by incorporating non-conductive regions of various sizes throughout the tissue (Tanaka et al., 2007; Burstein et al., 2009; Comtois and Nattel, 2011) (reparative fibrosis), either randomly throughout the atria, or based on imaging data. Endo-epicardial dissociation resulted (Eckstein et al., 2011) in a number of AF reentrant waves that was significantly higher than that in the case without dissociation, exacerbating AF complexity. The increase in collagen content in the interstitial spaces between fibers was not found to affect longitudinal conduction, (Burstein et al., 2009; McDowell et al., 2012, 2013) but caused slowed propagation in transverse direction, with the degree of slowing dependent of the length of the collagenous septa (Jacquemet and Henriquez, 2009).

**Figure 3:**
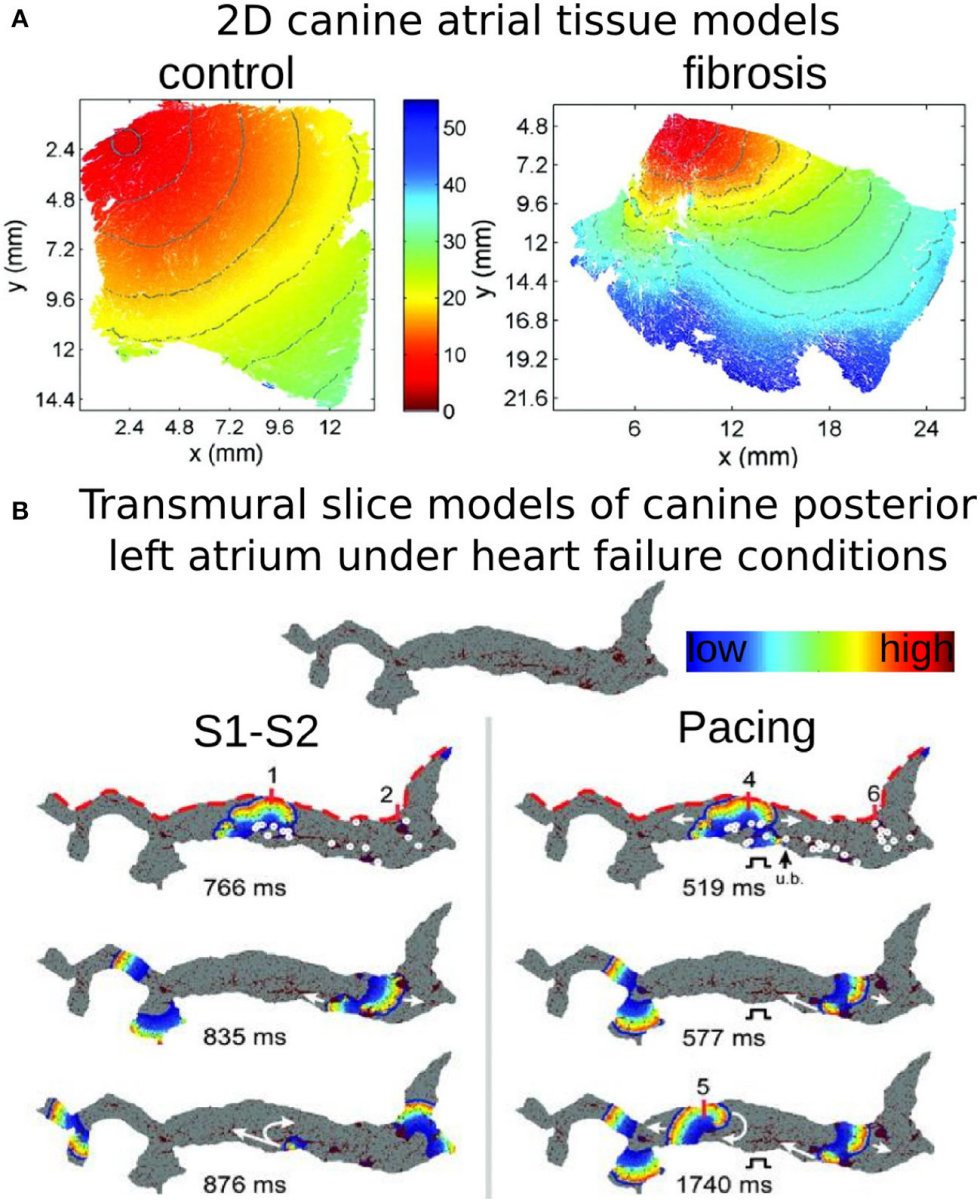
**Modeling fibrosis as regions of collagen presence**. Collagen is represented as an insulator. **(A)** Simulations of propagation in models of 2D tissue sections of canine atrium (control, left, and fibrosis, right). With permission from Burstein et al. (2009). **(B)** Simulations in models of a transmural slice of canine posterior LA under HF conditions; the top of the slice (red dashed lines) corresponds to the epicardial surface. Snapshots at several timeframes for cross-field stimulation (left), and pacing at a frequency of 6 Hz (right), as well as endocardial time-space plots. Colors indicate transmembrane voltage from low (blue) to high (red). The site of unidirectional block (ub). is indicated by a black arrow. White circles on the upper voltage maps indicate sites of wavebreak. With permission from Tanaka et al. (2007).

Atrial models incorporating transverse collagen deposition (Tanaka et al., 2007; Burstein et al., 2009; Comtois and Nattel, 2011) (as in reparative fibrosis) have highlighted the significant interruption and disarray in atrial conduction patterns caused by it. Importantly, collagen deposition rather than connexin-43 (Cx43) remodeling was found to be the major factor in atrial conduction disturbances under HF conditions (Burstein et al., 2009) (Figure 3A). Furthermore, it was established that not only the total amount, but also the specific spatial distribution of collagen deposition (e.g., as generated by a stochastic algorithm) governed the occurrences of conduction block (Comtois and Nattel, 2011). To evaluate the consequences of HF remodeling (ionic and structural) on AF dynamics in the posterior left atrium (LA), Tanaka et al. (Tanaka et al., 2007) used 2D models of transmural posterior LA sections generated from histological data; patchy distributions of collagen were also reconstructed from that data. Simulations demonstrated that whether the mechanism sustaining AF was reentrant or focal, fibrous patches of large size were the major factor responsible for the different dynamics of AF waves in failing vs. control hearts; they anchored reentrant circuits and impaired wave propagation to generate delays and signal fractionation (Figure 3B).

**Figure 4:**
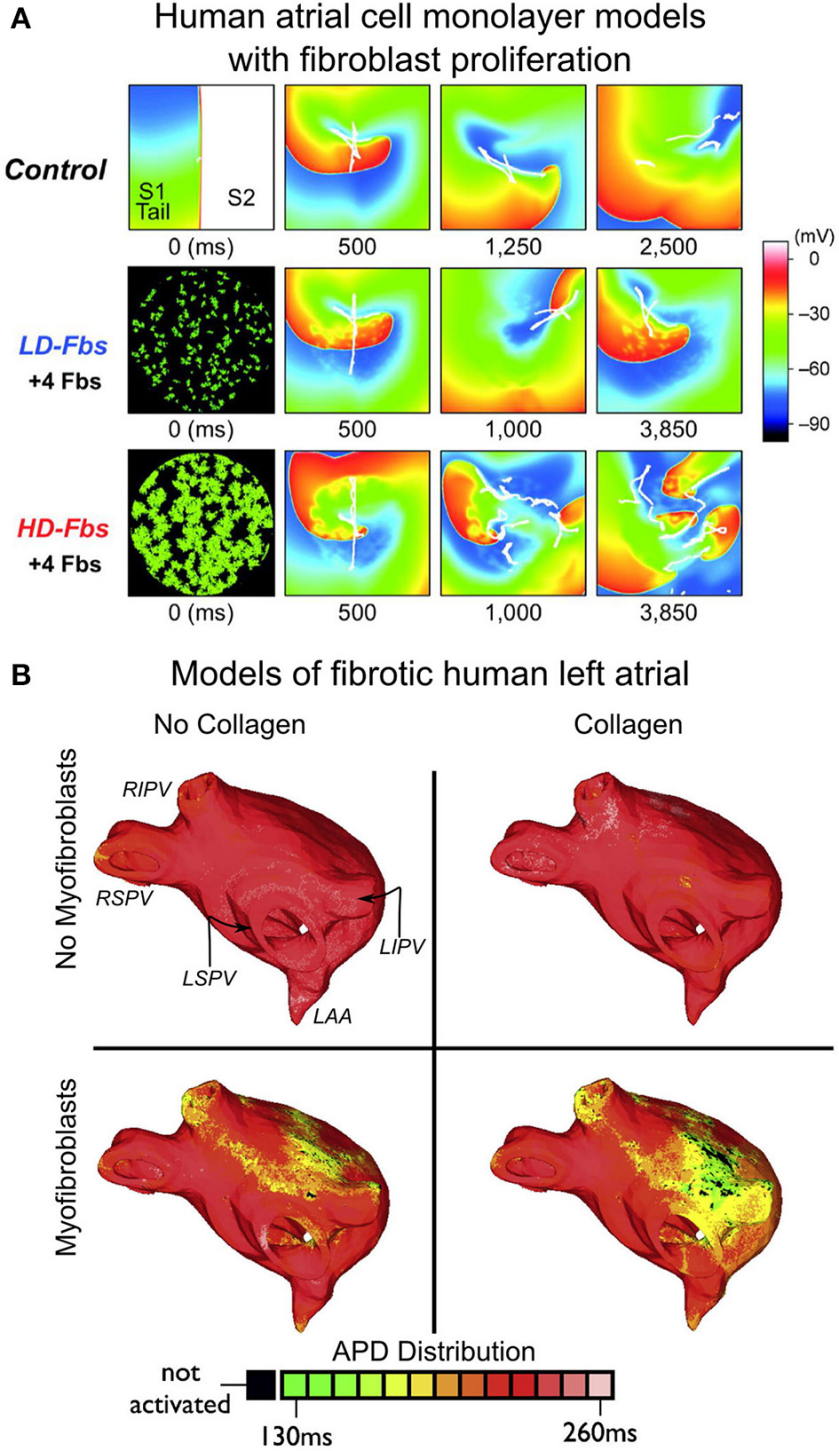
**Modeling fibroblast proliferation in the regions of fibrosis**. **(A)** Effect of myocyte-fibroblast coupling (modeled as in Maleckar et al., 2009a) on spiral wave behavior in a human atrial cell monolayer model of size 4.5 × 4.5 cm. Top, control case without fibroblasts. Middle and bottom, models of low-density and high-density fibroblast proliferation (LD-Fbs and HD-Fbs) in a central circular region of the sheet. In the LD-Fbs, and HD-Fbs models, atrial myocytes (100 pF), each connecting to 4 fibroblasts (6.3 pF) within the Fb-Area, account for 12.5% and 50.0% of that area, respectively. With permission from Ashihara et al. (2012). **(B)** Maps of action potential duration (APD) in four human atrial models (same atrial geometry). Fibrotic lesions are modeled with (bottom row), and without (top row) myofibroblast infiltration (and coupling to myocytes), as well as with (right column), and without (left column) diffuse collagen deposition for both sets of maps. Myofibroblasts in the fibrotic regions were coupled to atrial myocytes as described in Maleckar et al. (2009a) and Maleckar et al. (2009b). Anatomical landmarks in upper-left sub-panel: right inferior, right superior, left inferior, and left superior pulmonary veins (RIPV, RSPV, LIPV, LSPV, respectively); left atrial appendage (LAA). With permission from McDowell et al. (2013).

The third major component of fibrotic remodeling, fibroblast proliferation and phenotype switching, has also been represented in computational models of the atria, particularly in view of the fact that fibroblasts, in addition to being part of the structural remodeling of the atria, can also exert electrophysiological influences on neighboring myocytes, possibly either through electrical coupling (Camelliti et al., 2004), or via paracrine effects (Pedrotty et al., 2009). The first study to explicitly incorporate fibroblast presence as a representation of fibrotic remodeling was the 2D atrial model by Ashihara et al. (2012). Within the fibrotic region, coupling of fibroblasts (kinetics governed by a fibroblast ionic model) to atrial myocytes caused shorter action potential duration (APD), slower conduction, and lower excitability as well as spiral wave breakups. This effect was exacerbated when fibroblast density increased (Figure 4A). Interestingly, when fibroblasts were substituted by collagen in the model, wave breakups were not observed.

All three elements of fibrotic remodeling (gap-junction remodeling, collagen deposition, and myofibroblast proliferation), were combined together in the LA model generated from MRI-LGE data of a patient with permanent AF (McDowell et al., 2012, 2013), capturing accurately both the atrial geometry and the distribution of fibrotic lesions. The model was used to examine the mechanisms for AF initiation by pulmonary vein (PV) ectopic stimulation. The study found that for fibrotic lesions typical of human remodeled atria under the conditions of persistent AF, gap junction remodeling in the fibrotic lesions was a necessary but not sufficient condition for the development of AF following a PV ectopic beat. The sufficient condition was myofibroblast proliferation in these lesions, where myofibroblasts exerted either electrotonic, or paracrine influences on myocytes within the lesions. Deposition of collagen in the lesions assisted the myofibroblasts' paracrine, or electrotonic effects by additionally shortening APD there (Figure 4B). The electrophysiological representation of fibrotic remodeling in the human atrial models remains, however, controversial because of the lack of sufficient experimental data.

## Ischemic cardiomyopathy remodeling in the ventricles and its contribution to ventricular tachycardia

Understanding ventricular arrhythmia mechanisms for a variety of diseases involving regional changes in the passive electrical properties has been aided, to a significant degree, by models and simulations. For instance, models representing regional ischemia (Tice et al., 2007; Jie et al., 2008, 2010; Jie and Trayanova, 2010) characterized the substrate for ischemia phase 1B arrhythmias by examining how the interplay between different degrees of hyperkalemia in the surviving layers, and the level of cellular uncoupling between these and the mid-myocardium layers (i.e., change in the passive properties of the myocardium) combine with the specific geometry of the ischemic zone in the ventricles to result in reentrant arrhythmias.

Ischemic cardiomyopathy (i.e., infarct-related) VT is the most frequent clinical ventricular arrhythmia, present in 64% of patients with ventricular rhythm disorder and in 89% of patients with sudden cardiac death (Stevenson et al., 1985). Recently, the peri-infarct (border) zone surrounding the necrotic scar, also known as GZs, as indicated above, based on its appearance as a region of intermediate intensity in the LGE-MRI scans, has been shown to correlate with post-MI mortality (Yan et al., 2006), clinical VT (Roes et al., 2009), and VT inducibility (Schmidt et al., 2007). Histological studies have shown these GZ regions to be a heterogeneous mix of viable myocardium and necrotic scar (Arheden et al., 2000). Animal experimental evidence has implicated the GZ as the arrhythmogenic substrate in myocardial infarction (MI) (Estner et al., 2011); Ashikaga et al. demonstrated that in infarcted swine hearts, reentrant circuits were anchored to strands of viable myocardium positioned over intramural scars (Ashikaga et al., 2007).

A recent swine heart study (Ng et al., 2012) demonstrated the feasibility of using simulations to predict the existence of VT circuits. In another study (Pop et al., 2011), a correspondence between *in vivo* electroanatomical and *in silico* voltage maps was demonstrated using a model of infarcted pig ventricles reconstructed from *ex vivo* MRI, and DTMRI data. Their simulations in two infarcted hearts successfully predicted the VT-inducibility consistent with the *in vivo* electrophysiological studies.

**Figure 5:**
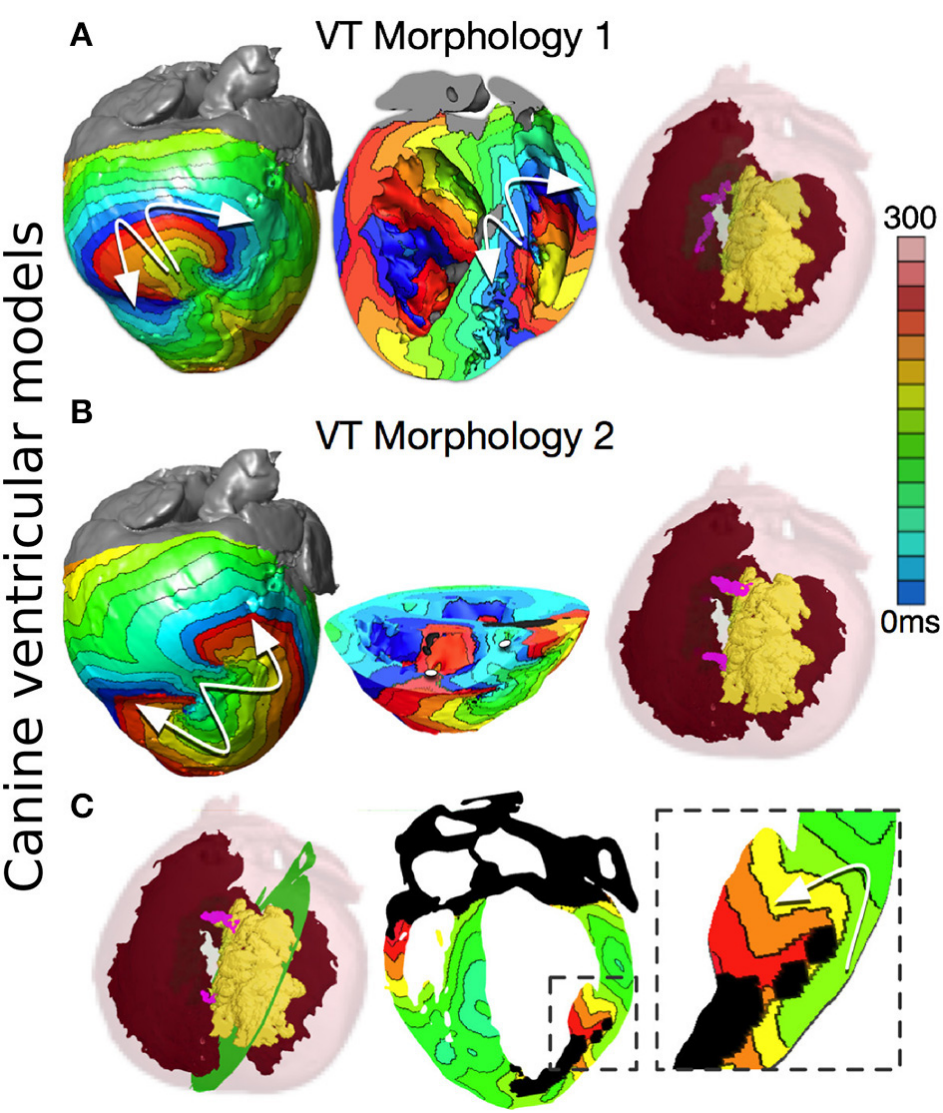
**Reentry morphologies during post-infarction ventricular tachycardia (VT) in a canine cardiac model**. **(A)** VT morphology 1: Activation maps on the epicardium and in a long-axis cross-section of the ventricles, revealing figure-of-eight reentries on the epicardium, and on the right ventricular (RV) endocardium. VT is sustained by two I-type filaments (pink lines) located within the GZ with endpoints on the epicardium and RV endocardium. (Red: endocardial and epicardial surfaces, Yellow: scar surface, semi-transparent green: GZ) **(B)** VT morphology 2: Activation maps on the epicardium and in a short-axis cross-section of the ventricles, revealing figure-of-eight reentry on the epicardium, and two breakthroughs on endocardium (white dots). Reentry was organized around two I-type filaments with endpoints on the epicardium and scar (pink lines). **(C)** Activation map showing an apparent reentry around a scar distal from filaments. The overall VT morphology is similar to VT morphology 2 in Panel **B**. With permission from Arevalo et al. (2013).

A clinically significant question is how infarct-related VTs relate to the specific GZ distribution and size in the ventricles. Addressing this question would provide an impetus to the development of improved criteria for stratifying arrhythmia risk in post-MI patients. A recent study (Arevalo et al., 2013) took this concept further and examined the role of infarct-related GZ extent in arrhythmogenesis, establishing that a minimum volume of remodeled tissue is needed for VT maintenance and demonstrating that the organizing center of infarct-related VT is located within the border zone, regardless of the pacing site from which VT is induced. An example of simulations of infarct-related VT using an MRI-based canine heart model (see Figure 1) is presented in Figure 5; the model incorporated experimental data on electrophysiological remodeling in GZ. Programmed stimulation from the endocardial surface in this model revealed conduction slowing in the GZ, giving rise to VT inducibility, and reentrant circuit morphology consistent with experimental data. There are two distinct VT morphologies in this infracted canine heart. The first VT morphology was a figure-of-eight pattern on the epicardium and right ventricular (RV) endocardium (Figure 5A). For this VT morphology, the reentry revolved around two I-type filaments (organizing centers of reentrant activity) with endpoints at the epicardium and RV endocardium (Figure 5A, pink lines). The filaments were fully contained within the GZ and the endpoints remained in the same locations for the duration of the VT. The second VT morphology was a figure-of-eight reentry on the epicardium, had a direction of rotation (chirality) opposite to that of the first VT morphology, and was manifested as breakthroughs on the LV, and RV endocardial surfaces (Figure 5B). This was due to the reentrant activity being organized around two I-type filaments with endpoints at the epicardium and the infarct scar (Figure 5B, pink lines). Since the filaments did not extend to the endocardium, no rotational activity was observed there. Both filaments were stably located within the GZ throughout the duration of the simulation. The intramural behavior of the reentrant circuits associated with the second VT morphology is presented in Figure 5C. Overall, the simulation results demonstrated that the organizing center of infarct-related VT is located within the GZ, regardless of the pacing site from which VT is induced. This result has important implications for ablation of infarct-related VT; it indicates that patient-specific simulations of VT could provide guidance for VT ablation in patients. Such simulation guidance could have a major clinical impact in predicting the optimal targets for catheter ablation of infarct-related VT in individual patient hearts.

**Figure 6:**
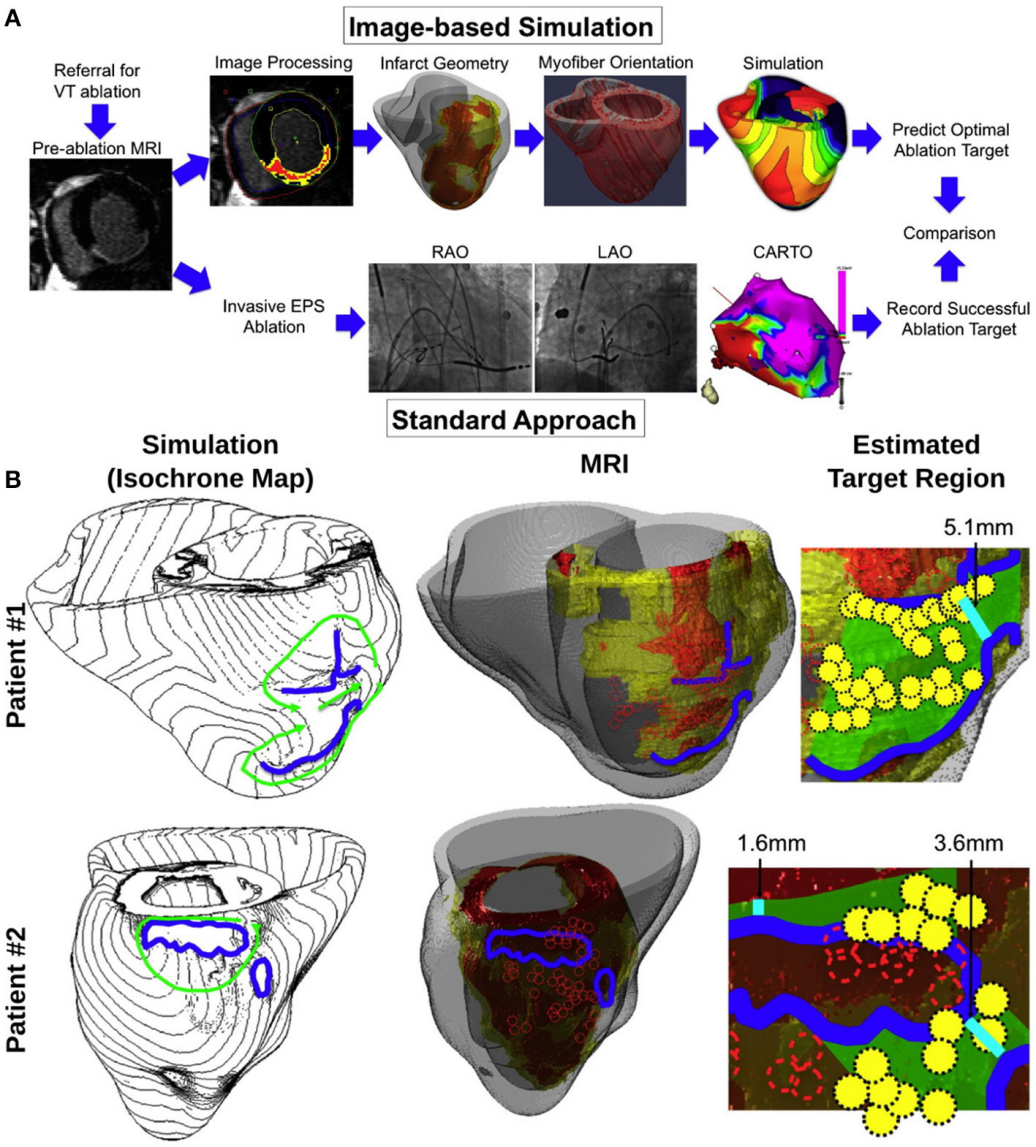
**(A)** Proposed simulation-guided approach for determining the ablation targets of infarct-related ventricular tachycardia. The patients referred for ventricular tachycardia (VT) ablation undergo pre-ablation MRI, which was processed to provide the heart and infarct geometry (scar: red; GZ: yellow). These geometrical data were incorporated into a model of VT to estimate potential target regions. This method is alternative to an invasive electrophysiology study (EPS), and ablation [“Standard Approach”; by using biplane X-ray fluoroscopy and electroanatomical mapping (CARTO)]. LAO—left anterior oblique; RAO—right anterior oblique. **(B)** Comparison between simulation-guided and standard electrophysiological approaches for identifying endocardial ablation targets in two patients with infarct-related ventricular tachycardias (VTs). Left column: propagation pathways (green) and lines of conduction block (blue) are overlaid over VT activation maps simulated in image-based patient heart models. Middle column: preablation infarct geometry (infarct scar: orange, border zone: yellow, and non-infarcted: gray) along with ablation lesions delivered by the standard approach (red circles), and conduction block lines as calculated from ventricular simulations. Right column: optimal ablation zones (green shading) predicted by simulations, with narrowest isthmuses indicated (cyan); in both cases, only a fraction of the ablation sites from the standard approach were within the predicted optimal LV endocardial ablation zone (yellow circles). Modified with permission from Ashikaga et al. (2013).

Another recent study (Ashikaga et al., 2013) was the first attempt to take this concept to the clinic. Figure 6A presents a schematic how computer simulation prediction of the optimal VT ablation targets could be used in lieu of invasive electroanatomical mapping of the ventricles. Figure 6B presents comparisons between simulation-guided and standard electrophysiological approaches for identifying ablation targets in two patients with infarct-related VTs. The results of the study demonstrated that that non-invasive simulation prediction of optimal targets for ablation of infarct-related VT could result in lesions that are much smaller than those executed in the clinic that successfully terminated VT (Figure 6B, right columns).

Simulation studies of ventricular arrhythmogenesis have recently begun to incorporate the role of cells other than myocytes in creating the arrhythmogenic substrate. Similar to the conditions under atrial fibrosis, differentiating fibroblasts, called myofibroblasts, have been reported to possibly play a role in the electrophysiological behavior of the scar, and GZ. Coupling to myofibroblasts in the region of infarct could affect cardiomyocyte electrophysiology, as demonstrated by simulation results (Maleckar et al., 2009a; Ashihara et al., 2012). A later study (McDowell et al., 2011) employed a high-resolution MRI-based computational model of the chronically infarcted rabbit ventricles, which was previously used (Rantner et al., 2012) to explore the role of infarct-related structural remodeling in vulnerability to electric shocks. The ventricular model was used to characterize the arrhythmogenic substrate resulting from myofibroblast infiltration (McDowell et al., 2011). It was found that myofibroblasts at low densities do not alter arrhythmia propensity, while at intermediate densities, myofibroblasts cause APD shortening, and exacerbate arrhythmia propensity. Interestingly, at high densities, myofibroblasts were found to protect against arrhythmia by causing resting depolarization, and blocking propagation. This study clearly indicated that non-myocyte cells could potentially have an important role in the altered passive electrophysiological properties of the myocardium.

## Concluding comments

The mechanisms that govern arrhythmia initiation and persistence in the heart, in both atria and ventricles, are highly complex, of dynamic nature, and involve interactions across multiple temporal and spatial scales, often leading to unpredictable outcomes and emergent phenomena. Electrophysiological experimental investigations in cells, tissues and the whole animal, and the human patient (Packer, 2004; Nattel et al., 2008; Iwasaki et al., 2011; Jalife, 2011; Schotten et al., 2011; Atienza et al., 2012) have led to a rapid increase in the body of knowledge regarding the mechanisms underlying arrhythmias. However, because of this inherent complexity, it is often difficult to dissect the contributions of individual players, and to elucidate interactions at a particular spatial scale. Computer simulations of cardiac electrophysiology have provided this ability, and this review article focuses, although not exhaustively and via examples from only a few simulation studies, on how remodeling in the “passive” electrical properties of the myocardium secondary to disease can render the organ susceptible to arrhythmias.

A lot remains to be uncovered, as remodeling of the electrical properties of the myocardium in disease, both passive and active, remains an intense area of research. As this review demonstrates, uncovering the integrative behavior of the heart resulting from such remodeling through simulation of atrial and ventricular function will continue to be strongly dependent on developments in experimental methodologies, which provide data to constrain, enrich, and validate the models. Of particular importance will be to fully characterize the complex remodeling that occurs in disease. Better understanding of the specific electrophysiological characteristics of remodeled tissue (i.e., GZ) surrounding the infarct in ischemic cardiomyopathy patients will enable more detailed mechanistic models of arrhythmia initiation, maintenance, and termination. Likewise, as experimental research addresses further the properties of the fibrotic myocardium, and in particular the electrophysiological aspects of the interaction between myocytes, and fibroblasts in the fibrotic lesions in the atria, atrial models are expected to provide a comprehensive view of the factors that drive arrhythmogenesis and influence arrhythmia dynamics in AF patients. Additionally, both atrial and ventricular modeling approaches could be improved by developing better capabilities to resolve the pathophysiological structure of the heart at high spatial resolution (isotropic voxel size of 1 mm or smaller); in particular, sensitive cardiac-specific tractography, and connectivity mapping techniques could provide a wealth of information valuable for constructing models of improved structural detail. Finally, present understanding of how atrial and ventricular myocardial fibers are oriented within the 3D volume of the heart is derived primarily from DT-MRI scans conducted on a handful of hearts *ex vivo*; imaging techniques capable of acquiring patient-specific maps of fiber orientation *in vivo* could be used to validate atlas-based approximations and incorporate disease-related changes in fibrous structure.

Clearly, we are poised at an exciting moment in cardiovascular medicine. The findings of molecular biology of the heart, the emergence of new technologies for measuring the properties of cells, tissues, and organ function, and the impact of Moore's law on computational modeling are coming together to drive the creation of new, quantitative, model-based approaches to understanding the function of the heart in disease, and to cardiovascular medicine of the future.

### Conflict of interest statement

The authors declare that the research was conducted in the absence of any commercial or financial relationships that could be construed as a potential conflict of interest.
